# Influence of Air Flow on Luminescence Quenching in Polymer Films towards Explosives Detection Using Drones

**DOI:** 10.3390/polym14030483

**Published:** 2022-01-25

**Authors:** Daegwon Noh, Emmanuel K. Ampadu, Eunsoon Oh

**Affiliations:** 1 Department of Physics, Chungnam National University, 99 Daehak-ro Yuseong-gu, Daejeon 34134, Korea; fo1109@cnu.ac.kr (D.N.); ekampadu@cnu.ac.kr (E.K.A.); 2 Institute of Quantum Systems (IQS), Chungnam National University, 99 Daehak-ro Yuseong-gu, Daejeon 34134, Korea

**Keywords:** conjugated polymer, explosives detection, computational fluid dynamics, photoluminescence

## Abstract

Explosive detection has become an increased priority in recent years for homeland security and counter-terrorism applications. Although drones may not be able to pinpoint the exact location of the landmines and explosives, the identification of the explosive vapor present in the surrounding air provides significant information and comfort to the personnel and explosives removal equipment operators. Several optical methods, such as the luminescence quenching of fluorescent polymers, have been used for explosive detection. In order to utilize sensing technique via unmanned vehicles or drones, it is very important to study how the air flow affects the luminescence quenching. We investigated the effects of air flow on the quenching efficiency of Poly(2,5-di(2′-ethylhexyl)-1,4-ethynylene) (PEE) by TNT molecules. We treated the TNT molecules incorporated into the polymer film as non-radiative recombination centers, and found that the time derivative of the non-radiative recombination rates was greater with faster air flows. Our investigations show that relatively high air flow into an optical sensing part is crucial to achieving fast PL quenching. We also found that a “continuous light excitation” condition during the exposure of TNT vapor greatly influences the PL quenching.

## 1. Introduction

Detecting the presence of explosives in the surrounding environment, whether in the battle field or in the fight against terrorism, is (increasingly) critical to saving lives and property. The majority of casualties in modern and urban warfare are from the failed detection of improvised explosive devices (IED) [1,2,3,4]. Many present and past conflict zones are still littered with millions of landmines, continuing to bring havoc to the local populations, as well as preventing the further advancement of these mostly under-developed regions and nations [5,6]. Homeland security and border control spend an enormous amount on explosive material screening and detection [7], bringing various levels of discomfiture to passing passengers or travelers. The basic science and technologies used to enable such detection have been known for a few decades, with researchers seeking effective non-contact detection devices. However, the meaningful application of such technologies in widely available devices remains a very elusive task.

Explosive materials often contain nitro compounds, such as nitroaromatics (TNT), nitramines (RDX), and nitrate esters (PETN) [8,9]. Various detection methods of these compounds, including ion mobility spectrometers, surface enhanced Raman spectroscopy, and luminescence-based techniques, have been developed [10,11,12,13,14]. Among these methods, luminescence-based methods utilizing the reduction in photoluminescence (PL) intensity produced by sensing materials (known as fluorescence quenching) using nitro-containing explosive vapors have been extensively studied after the pioneering work done by Yang and Swager in 1998, who used conjugated polymers for the sensing of explosives [15]. As a typical exciton lifetime is on the order of ~ns, in this paper, we will use the term photoluminescence (PL) rather than fluorescence.

A Korean company, PNL global, initiated a “drone” project in order to detect landmines, as the PL quenching method requires relatively little electric power consumption and the equipment can be lighter compared to other sensing systems. In the case of Raman-based sensing, for example, a laser is used as an excitation light source, whereas an LED (light-emitting diode) can be used for luminescence quenching; this difference is important for the flight time of drones. Although drone flying may not be able to pinpoint the exact location of the landmines and explosives, the identification of the explosive vapor present in the surrounding air provides significant information and comfort to the personnel and explosives removal equipment operators. In this system, the speed of the drone will naturally direct the nitro-containing air flow into the sensing compartment.

In 2005, Zhao and Swager compared the luminescence quenching of PPE and PPD polymers in solutions and in films, and discovered that the response behaviors in the solid states are governed by different factors than those in solutions [16]. For real-time landmine sensing, it is necessary to detect nitro-containing vapors using film-type sensors. Despite the enormous progress made in sensing polymers, it is difficult to find any literature regarding the effect of air flow in PL quenching, as most gas-phase PL quenching experiments have been carried out in quartz vessels without air flow [1,2,6,9,15,17,18,19,20,21,22,23,24]. In order to utilize a sensing technique employing unmanned vehicles or in drones, it is very important to study how the air flow affects PL quenching. In this paper, we discuss how the air flow containing TNT vapors affects the PL quenching (PQ) efficiency in polymer films. We also discuss how the exposure of excitation light influences PQ. Our study provides information on the theoretical understanding of molecular diffusion from air into a polymer film.

## 2. Materials and Methods

### 2.1. Film Fabrication

Poly(2,5-di(2′-ethylhexyl)-1,4-ethynylene) (PEE) powders were purchased from Sigma Aldrich(St. Louis, MO, USA). It is well known that the solvent is an important parameter affecting the degree of aggregation and the nature of chain conformation in the final film formation, which can influence the optical properties of the films [25]. We tested acetone, ethanol and toluene for use as the solvent in PEE polymer. Among the solvents tested, PEE polymer was successfully dissolved only in toluene up to 10 g/L concentration. The solution was sonicated for ~10 min to ensure a uniform mixture. The PEE thin films were fabricated on 1 cm × 1 cm microscope slide glass. An extensive cleaning protocol, described below, was followed to prepare glass substrates for film deposition to get rid of all the particles and organic residues on the surface. The glass substrates were first wiped with ethanol. The substrates were sonicated for 10 min each in acetone, in isopropyl alcohol and in ethanol. The substrates were then dried by blowing nitrogen gas.

PEE polymer films were deposited on the cleaned glass substrates by spin-coating 20 µL PEE/toluene solution at 3000 RPM (revolutions per minute) for 60 s. For thicker (very thin) films, 20 µL of 10 g/L (0.01 g/L) was spin-coated at 2000 RPM (3000 RPM) on the cleaned glass substrates. After the spin-coating, the films were thermally annealed on a hot plate for ~1 min at 100 °C in order to ensure the complete evaporation of toluene. The annealed samples were then stored in a tray and covered with aluminum foil. The foil was used to protect the photo-degradation of the films. We found that the vacuum sealing of the aluminum-covered tray containing the polymer films prevented possible degradation under atmospheric conditions.

The interaction between polymers and metal was studied earlier in Refs. [26,27,28]. We spin-coated the PEE solution onto various metals and semiconductors, such as Au, Zn, Ti, TiO_2_, FTO, ZnO and meshed Ni substrates, in order to observe the effects of substrates. Among the various substrates that we tried, the quenching efficiency was improved only on Zn substrates. For the deposition of PEE thin film onto Zn foil, the Zn foil was attached on a glass slide to prevent bending or folding during spin-coating. The surface morphology of PEE polymer on glass was determined using a cold type field emission scanning electron microscope (FESEM, S-4800, Hitachi High-Technologies, Tokyo, Japan and Merlin Compact, Carl Zeiss, Jena, Germany). A uniform morphology was observed from the surfaces of films, and the thickness of the film deposited with 1 g/L solution was estimated to be ~40 nm.

### 2.2. PL Quenching Set-Up

**Figure 1:**
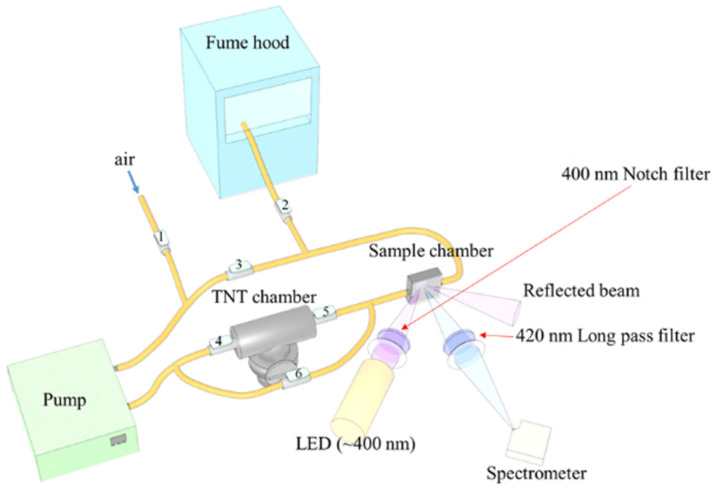
Experimental set-up. A closed flow cycle system was used to maintain a constant vapor pressure and to ensure personnel safety.

A custom-built unit was used as shown in Figure 1. The set-up was made up of a TNT chamber, a sample chamber covered by a transparent sapphire window, a pump, Teflon, and silicon tubes, as well as valves that were manually opened and closed during measurements. The flow rate of the pump was controlled with a computer. During PL quenching measurements, valves 1, 2 and 6 were closed while the rest were opened to allow the flow of TNT vapor in a closed cycle. To maintain a relatively constant vapor pressure, a closed cycle was used. After the PL quenching measurement, valves 3, 4 and 5 were closed, and all the other valves were opened so that the air could be pumped into the sample chamber and residual TNT molecules could flow into a fume hood. A FLAME-S-VIS-NIR-ES (FLAME miniature spectrometer, Ocean Insight^®^, Orlando, FL, USA) was employed to measure the PL spectra. The polymer films were illuminated with an LED (~400 nm) operating in a pulse mode. Pulse mode was used to decrease the effects of optical degradation, which may be caused by the CW mode and highpower LED excitations. All the optics as well as the sample chamber were completely covered with a dark curtain to block out any external light during measurements.

## 3. Results and Discussion

### 3.1. Spectral Analysis

**Figure 2:**
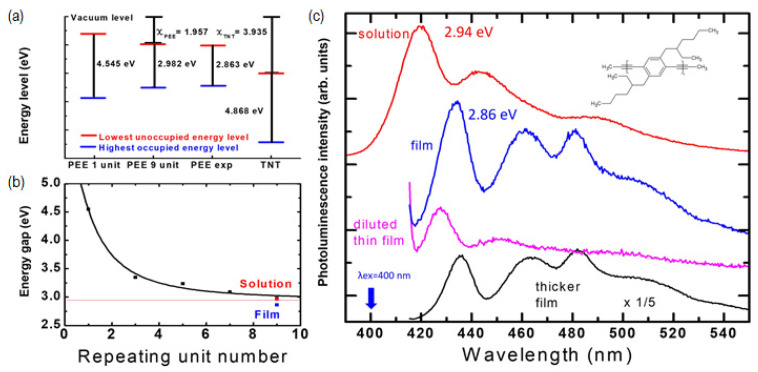
(**a**) The HOMO and LUMO energy levels of PEE and TNT. Calculations were done with the Orca ab-initio calculation package with the DFT-B3LYP/6-31G level of theory. (**b**) Energy gap as a function of the number of PEE monomer repeating units. Red and blue dots are the experimentally observed PL peak energies in solution and in film, respectively. (**c**) PL spectra of PEE in toluene solution phase (red), film phase (blue), thin film phase (magenta), and thicker film phase (black). The molecular structure of PEE is shown in the inset.

In this section, we discuss the bandgap energies and the PL spectra of PEE polymer. The electronic delocalization of conjugated polymers was discussed earlier [29,30,31], and HOMO LUMO levels were calculated by Density Functional Theory (DFT) simulations for some conjugated polymers [32,33]. In Figure 2a, we show the HOMO and LUMO energy levels and bandgap energies of the PEE polymer obtained from DFT simulations (Orca 4.2.0) using the B3LYP method with a 6-31G basis function. In the PEE polymer that we used, the attached side chains were slightly different from the PPE polymers previously studied [15,19,34,35,36]. Figure 2b shows the decrease in the theoretically expected bandgap of the polymer as the number of repeating units increased from 1 to 9, reflecting the increased delocalization of the electronic wave function. The result fits well with the formula [37] $E_{\left(n\right)}=E_{0}+2\beta cos\left(\frac{\pi }{n+1}\right)$, with $E_{0}$ = 4.54 eV and β = 0.79 eV, where *n* is the number of repeating units.

Figure 2c shows the PL spectra of the PEE polymer in the solution phase (red) as well as in the solid film (blue, magenta, black) with an excitation wavelength of 400 nm. The PL peak is seen at ~432 nm (2.86 eV) in film and at ~420 nm (2.95 eV) in a (toluene) solution form. The experimentally observed value of the PL peak energy agrees reasonably well with the bandgap energy predicted from the DFT simulation with nine repeating units (2.98 eV) (see Figure 2b). Although our calculations did not take into account the conformation change of polymer, the variation in the conformation may also affect the electronic wave function and bandgap.

**Figure 3:**
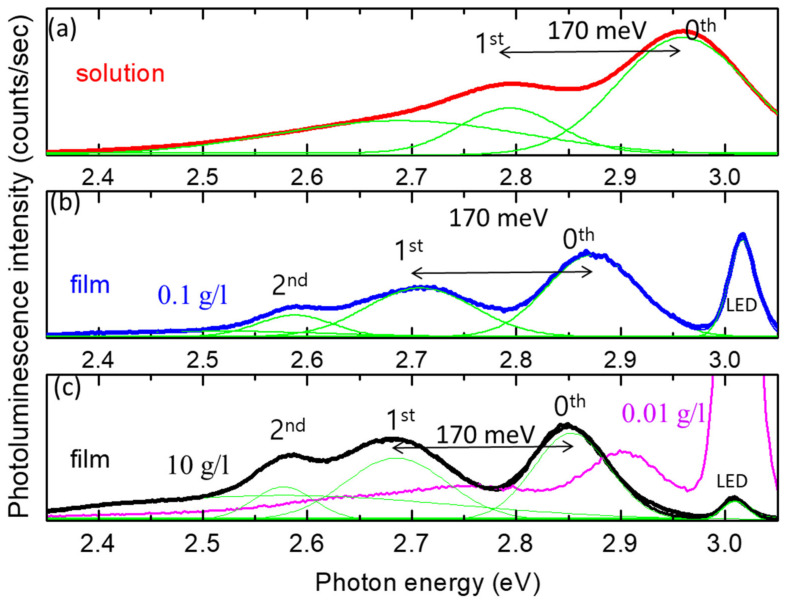
PL spectra of PEE with toluene solvent. The spectra were divided by spectrometer sensitivity. In the spectra, the main PL peak (labeled 0th) corresponds to the bandgap energy and the PL peaks (labeled lst and 2nd) are attributed to the phonon side-band peaks. Green curves show the peak deconvolution results with Gaussian functions. (**a**) Solution phase of PEE in a concentration of 0.1 g/L. (**b**) PEE film fabricated with a concentration of 0.1 g/L. (**c**) PEE films fabricated with concentrations of 10 g/L (black) and 0.01 g/L (magenta).

In Figure 3, we show the normalized PL spectra of the film and solution as a function of photon energy (eV) rather than the wavelength (nm). In Figure 3, we divide the PL spectra shown in Figure 2c by the CCD spectral sensitivity (Sony ILX511B linear silicon CCD array), and resolve the modified PL spectra into several Gaussian peaks. The small peak at ~3.0 eV originated from the reflected/scattered light of the 400 nm excitation beam. As seen in Figure 3a,b, the energy difference between the main PL peak (labeled 0th) and the closest phonon side-band peak for both solid and solution (labeled 1st) was about 170 meV, corresponding to a phonon energy of 1400 cm^−1^ [38,39,40]. We also performed micro-Raman measurements of our polymer film. In order to obtain a sufficient Raman signal from our polymer film, we made a thick film and confirmed a broad Raman peak at around 1400 cm^−1^, as shown in Supplementary Figure S1.

In Figure 3c, we show the PL spectra of the films spin-coated with 10 g/L (0.01 g/L) toluene solution, where the film is thicker at higher concentrations of solution. The red shift (blue shift) of the 0th PL peak energy can be observed for thicker (thinner) film. The thickness-dependent PL peak shift and relative peak intensities are very similar to those in the case of MEH-PPV [41]. The authors mentioned that the conformations of molecular chains and the stacking of the chains in films varied with film thickness, and were different from those in solutions. We note here that the relative peak intensities of the side bands (first and second) with respect to the main peak (0th) appear to be smaller for thinner films and larger for thicker films. A detailed discussion of the origin of the variation in the PL spectra is out of the scope of this paper, but we would like to mention that the quenching efficiencies of these thinner and thicker films were found to be lower.

### 3.2. PL Quenching Results

We employed the PEE polymer for use as an explosive sensing material based on PL quenching. It has been reported that the PL quenching of conjugated polymer by nitro-based explosives is mainly due to the photo-electron transfer, whereby the photo-excited electrons (excitons) in the LUMO states transfer to the lower-lying energy levels of the analyte molecules [5,9,13,15,31]. As seen in Figure 2a, the electron affinity of the PEE polymer is smaller than that of TNT, allowing the photo-electron to transfer to TNT molecules. We tested the PL quenching observed with both the solution (TNT/DI water) and vapor phases of TNT. We expect the vapor pressure of TNT will be reduced to about one-third of the equilibrium vapor pressure of TNT, which is known to be 7 ppb at 25 °C [13,42,43], considering the ratio of the TNT chamber volume and the total volume of the closed cycle system, including the tubes and sample chamber.

**Figure 4:**
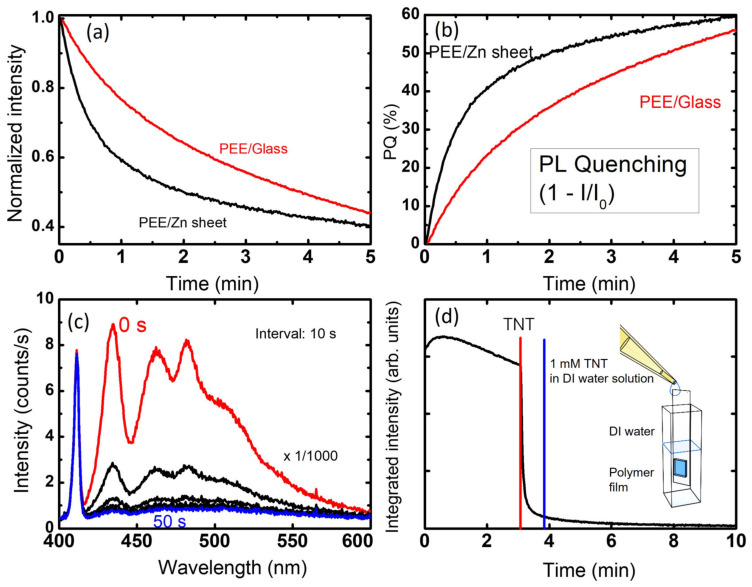
(**a**) Time-dependent integrated PL intensity of PEE films deposited on glass and on Zn sheet when exposed to TNT vapor at a flow rate of 6 L/min. (**b**) PL quenching curves of PEE films by TNT vapor as a function of time. (**c**) The change in the PL spectra observed with a glass/PEE film, which was initially placed in a transparent glass cuvette containing DI water. When 1 mM aqueous TNT solution was added, PL quenching was observed. (**d**) Time-dependent integrated intensity curve of PEE film deposited on glass and placed in aqueous solution. The arrow in the figure indicates the time when the TNT solution was added. A schematic diagram of the experimental set-up is shown in the inset.

In Figure 4a, we show the integrated PL intensity as a function of time after the TNT vapor exposure with a 6 L/min flow rate, corresponding to the air drift velocity of 3 m/s. Figure 4b shows the PL quenching efficiency PQ = (I_0_ − I)/I_0_, where I_0_ and I are the PL intensities before and after TNT vapor exposure. The quenching efficiency was ~23% (~41%) after 1 min and ~44% (~55%) after 3 min for glass (Zn) substrates, respectively.

The PQ values obtained with Zn substrates were found to be somewhat better than those on glass substrates. One possibility is that the improved PQ is due to the surface roughness. A planar SEM image of the Zn substrates depicting the surface roughness is shown in Supplementary Figure S2. The gas reaction is supposed to increase with increasing surface to volume ratio. Yang and Swager [15] utilized three-dimensional pentiptycene moieties to make polymer films with a porous structure. The lower PQ values that we obtained were probably due to the planar structure of PEE polymer, which could be partially overcome by the increased surface to volume ratio in Zn substrates. However, no improvement was observed with other rough substrates, such as rough sapphire substrates. This may be simply because the films deposited on rough substrates often induce aggregation conformation. Since the work function of Zn is 4.3 eV, it is difficult to assume that electrons are provided by the Zn metal substrates. Due to the reflection of the excitation light on the metal surface, the PL intensity was 2–3 times greater compared to the film deposited on glass under identical conditions. Although the quenching efficiency was better with the film on Zn substrates, in this paper, we mostly focus on the results derived with glass substrates, since the reproducibility and uniformity were better on glass substrates.

In Figure 4c, we show the change in the PL spectra with TNT solution after every 10 s. Figure 4d shows the integrated intensity as a function of time. For the solution phase test, we used a glass cuvette and slide glass block to measure the PL change. The PL from the polymer film coated on glass was measured while immersed in DI water contained in a glass cuvette. The film was excited with 400 nm LED light operating in pulse mode. After ~3 min of excitation, 0.2 mL of 1 mM TNT/DI water solution was added into the 1.8 mL of DI water, and the quenching of PL intensity was observed. The schematic of the experimental set-up is shown in the inset of Figure 4d.

### 3.3. Flow Dependence of PL Quenching

**Figure 5:**
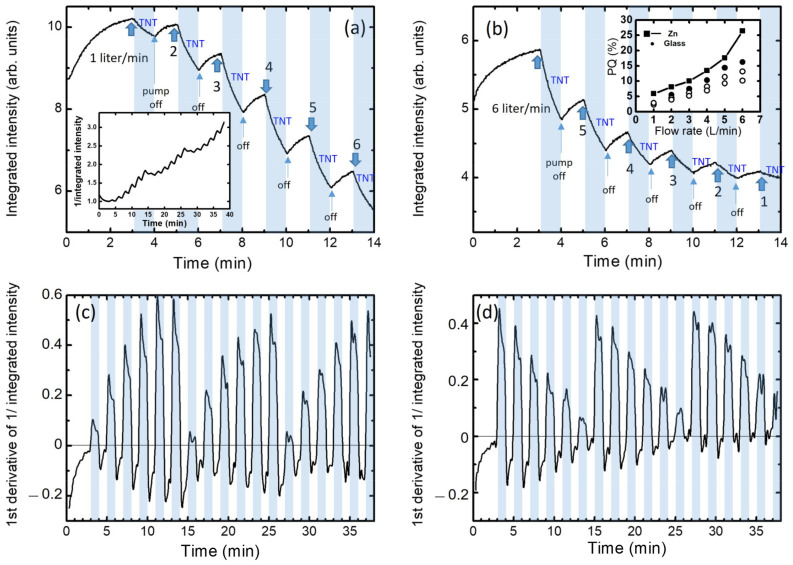
Time-dependent integrated PL intensity with various flow rates observed with glass/PEE films. The flow rate was controlled with a pump, as shown in Figure 1. The pump was turned on and off for a minute for each flow rate. A closed cycle was used while the TNT vapor was already inside of the sample chamber. Blue shaded parts indicate the pump-on period. The flow rates were changed (**a**) from 1 L/min to 6 L/min and (**b**) from 6 L/min to 1 L/min. The cycle was repeated 3 times and the inset of (**a**) shows the reciprocal of the normalized intensity. The inset of (**b**) shows the PQ (%) as a function of flow rates for the repeated cycles. The PQ from Zn/PEE film is also included. (**c**,**d**) represent the first derivatives of the reciprocal normalized integrated intensities of (**a**,**b**).

In order to investigate the flow dependence of PQ (PL quenching), the PL intensity was monitored with controlled air flow rates (Figure 5a). Prior to the measurements, TNT vapor was initially contained inside of a TNT chamber, and a valve in the TNT chamber was closed so that only TNT vapor, not TNT powder, could circulate through the sample chamber (see Figure 1). The air flow was controlled by the pumping speed. For the first 3 min, the air flow was maintained at zero, and the PL intensity was found to be slightly increased. The increase in PL under light exposure was observed earlier in MEH-PPV polymers, and was attributed to the planarization of polymer chains by light [44,45]. After 3 min, we turned on the pump to circulate the air containing the TNT vapor inside of the closed system for 1 min. PL quenching was clearly observed as we turned on the pump. We then turned off the pump, so that there was no air flow for the next 1 min. The arrows in the plots represent when the pump was turned on and off.

Although the TNT vapor remained in the sample chamber, the PL intensity remained slightly increasing when the pump was turned off. We continued this process with a flow rate increasing from 1 L/min to 6 L/min (Figure 5a). We note here that we circulated the TNT vapor for 30 s before we started this measurement, so that the PL intensity was not affected by the initial unstable change in the TNT vapor pressure in the sample chamber. Considering the total volume of the air hoses and sample chamber with respect to the volume of the TNT chamber, the stabilized vapor pressure in the sample chamber was expected to be one-third of the equilibrium TNT vapor pressure at 25 °C. As seen in the inset of Figure 5a,b, it is very clear that the quenching efficiency was greater with a faster air flow.

We then repeated the process with a decreasing flow rate from 6 L/min to 1 L/min three times, and the PQ during the first minute of TNT containing air flow is shown in the inset of Figure 5b. Again, the PQ was observed to be larger with a larger air flow, and even with a decreasing air flow. Same experimental procedures were followed using polymer films deposited on Zn substrates. The PQ values derived with the Zn substrate are included in the inset of Figure 5b.

In the inset of Figure 5a, we show the reciprocal intensity of the flow-dependent PL intensity change. When the TNT molecules are attached on the surface or diffused into the film, the decrease in PL intensity by the photo-electron transfer or by the energy transfer can be considered as caused by the increase in the non-radiative recombination rate $k_{nr}\left(t\right)$. From the internal quantum efficiency (*η*) of the polymer film,

$$\eta =\frac{Number\;of\;emission\;photons}{Number\;of\;absorption\;photons}$$

$$\eta \left(t\right)=\frac{k_{r}}{k_{r}+k_{nr}\left(t\right)}$$

where $k_{r}$ is radiative recombination rate.

Then we obtain

$$\frac{\eta_{0}}{\eta \left(t\right)}\left(=\frac{I_{0}}{I\left(t\right)}\right)=\frac{k_{r}+k_{nr}\left(t\right)}{k_{r}+k_{nr0}}$$

$$\frac{d}{dt}\frac{\eta_{0}}{\eta \left(t\right)}\left(=\frac{d}{dt}\frac{I_{0}}{I\left(t\right)}\right)=\frac{1}{k_{r}+k_{nr0}}\frac{d}{dt}k_{nr}\left(t\right)$$

Here, $k_{nr0}$ is the initial non-radiative recombination rate, and $\eta_{0}$ is the internal quantum efficiency without TNT molecules. We assume that $k_{r}$ is independent of time. Figure 5c,d show the first derivatives of the reciprocal PL intensity ($\frac{I_{0}}{I\left(t\right)}$) obtained from the raw data in Figure 5a,b, respectively. The term $\frac{d}{dt}k_{nr}\left(t\right)$ represents how rapidly the non-radiative recombination rate increases as TNT molecules are attached on the surface or diffused into the film.

**Figure 6:**
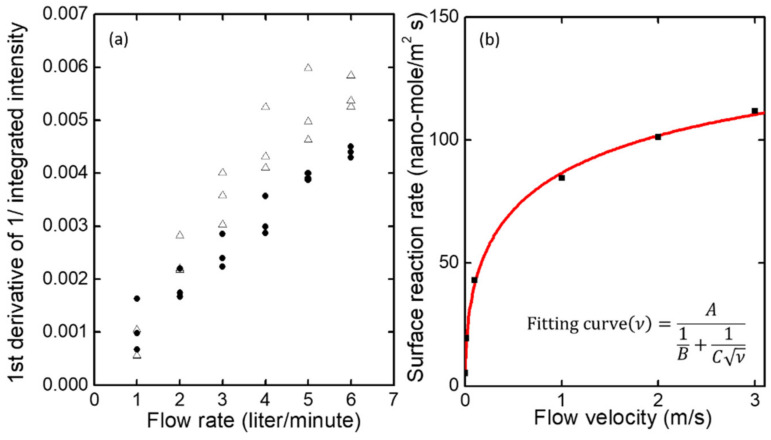
(**a**) The peak values of the first derivatives of the reciprocal normalized integrated intensities obtained from Figure 5c,d as a function of flow rate. The first derivative represents how rapidly the non-radiative recombination rate increases as TNT molecules are attached on the surface or diffused into the film. The effect of flow rate on the first derivative is clearly observed. (**b**) Effect of flow velocity on surface reaction rate. The black points are those obtained from CFD simulation. A fitting curve is shown in red. The fitting formula is included.

As seen in Figure 6a, the $\frac{d}{dt}\frac{I_{0}}{I\left(t\right)}$ values become larger with increasing flow rates. Since TNT molecules act as non-radiative recombination centers, the increase in $\frac{d}{dt}k_{nr}\left(t\right)$ with air flow can be attributed to the increased mass transport of TNT molecules onto the polymer film under a fast flow rate.

In order to understand mass transport as a function of air flow, we performed CFD (computational fluid dynamics) simulation. For the CFD simulation, we assumed that the TNT molecules would react on the sample’s surface with some specific reaction constant. In Figure 6b, we show the CFD simulation result of the surface reaction rate as a function of the air flow. Detailed simulation parameters and conditions are listed in Table 1. As seen, the surface reaction rate may strongly depend on air flow, but the surface reaction rate predicted by the CFD simulation tends to become saturated with flow. The CFD model does not account for the chemical binding interaction or electrostatic force between the molecules and the polymer’s surface. In the PL quenching process, the importance of the binding strength between the sensing polymer and analytes has already been suggested [13,15,46,47]. As the CFD simulation predicts that the diffusion of TNT molecules is affected by the flow rate, we suspect that some other factors, such as inter-molecular forces and photochemical interactions, may also affect the flow rate dependence of PL quenching.

### 3.4. Light Illumination Effect

**Figure 7:**
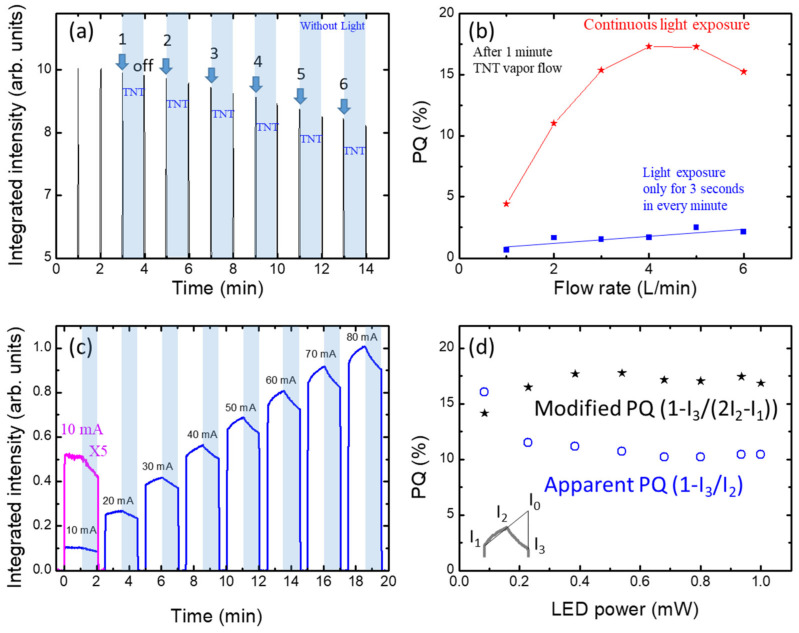
(**a**) Time-dependent integrated PL intensity with various flow rates observed with glass/PEE films. LED illumination was only for 3 s. Blue shaded parts indicate the pump-on period and the arrows indicate the flow rate change. (**b**) PQ values calculated from the raw data obtained from (**a**). The PQ values were compared with the raw data from Figure 5a and the effect of continuous LED illumination was observed. (**c**) Integrated intensity of glass/PEE film with various LED powers. LED currents are indicated. Blue shaded parts indicate the pump-on period with 6 L/min flow rate. (**d**) The PQ values were obtained from (**c**). Modified PQ (1 − I_3_/(2I_2_ − I_1_)) calculated based on the assumption that the intensity increases linearly with time.

In an effort to investigate possible factors that affect the quenching efficiency, we studied the influence of the excitation light. During the measurements shown in Figure 7a, we shined the LED excitation light for only three seconds every 1 min, rather than using continuous light exposure. As expected, the PL intensity was zero when there was no light exposure. After 3 min, the pump was turned on and TNT vapor flowed at 1 L/min for one minute, and the PL intensity was measured only during the short exposure time. Then, the pump was turned off for the next minute and the PL intensity was recorded again. The experimental process was identical to that used for Figure 5a,b, except that the light exposure was only three seconds every minute. In Figure 7b, we compare the quenching efficiencies obtained from the raw data shown in Figure 5a. As seen, the PQ was reduced to about one-fifth, compared to the case of continuous light exposure. To our knowledge, there has been no report on the effect of “continuous light exposure” on PL quenching efficiency. One possible cause may be the interaction between excitons and phonons; the binding of TNT molecules with a polymer film gets easier as the atoms constituting the polymers vibrate. Another possibility is that the planarization of polymer chains by light helps molecule transport. In any case, LED light is necessary not only for the excitation of the PL measurements, but also for the interaction of polymers and TNT molecules.

We investigated the excitation power dependence and have shown the results in Figure 7c. As seen in Figure 7d, the PQ values (blue) appeared somewhat decreased when increasing the excitation power up to the LED power of 0.4 mW, above which the PQ was almost independent of the LED power. As in Figure 5a, the air flow was zero for one minute, and then the pump was turned on so that the air flow (containing TNT vapor) was 6 L/min for the next one minute. We note that the PQ values in Figure 7d were obtained without taking into account the increase in PL due to the LED excitation. Considering the increase in PL intensity with the exposure of the light, we used I_0_ = I_2_ + (I_2_ − I_1_) rather than I_0_ = I_2_, where I_1_ was the recorded PL intensity when the light was turned on and I_2_ was the PL intensity just before TNT vapor exposure. Here we assumed that the PL intensity would linearly increase during the light exposure. The quenching efficiency was thus calculated using the relation (I_0_ − I_3_ = (1 − I_3_/(2I_2_ − I_1_)), where I_3_ is the PL intensity after quenching. We have included the calculated “modified PQ” values in Figure 7d. Above 0.4 mW, the PQ was found to be almost independent of the LED power. 

**Figure 8:**
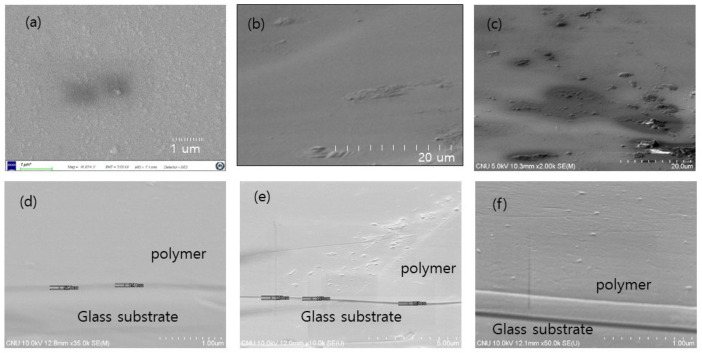
SEM images of polymer films. (**a**) Fresh PEE film morphology deposited with 10 g/L concentration. (**b**) Fresh film with 0.1 g/L concentration. (**c**) Film with 0.1 g/L concentration. (**d**–**f**) SEM images of the used polymer films obtained from PNL global company. In order to observe the morphologies more clearly, the samples were slightly tilted for SEM measurements.

In addition to luminescence quenching, the change in surface morphologies was also investigated. Figure 8 shows the planar SEM images of the as-deposited polymer films and those of the films after exposure to TNT molecules. In Figure 8c, it appears that TNT exposure led to the formation of localized swelling on the surfaces of the films.

## 4. Conclusions

We investigated the air flow dependence of quenching efficiency using TNT molecules for future drone applications. In order to maintain a relatively constant vapor pressure of TNT molecules, the vapors were circulated in a closed cycle. CFD (computational fluid dynamics) simulation showed that the mass transport of TNT molecules can be enhanced with air flow under sufficiently high reaction conditions. The photo-electron transfer process from the polymer to the TNT molecules probably involves an electro-static force, which can be assisted by surface friction. This may imply that turbulence flow helps the PL quenching process, while an ideal laminar flow was assumed in the CFD simulation. It was found that PL quenching was somewhat increased with the polymer film deposited on the Zn sheet with a rough surface. We also found that “continuous light excitation” during the exposure of TNT vapor greatly influences PL quenching.

We treated the TNT molecules attached on the surface or diffused into the film as non-radiative recombination centers, similar to carrier trapping impurities in semiconductors, and the PL quenching was associated with the increase in non-radiative recombination rate. From the time-dependence of the reciprocal PL intensity, we found that $\frac{d}{dt}k_{nr}\left(t\right)$ was greater with faster air flows. For drone applications, a flow-guiding device is used to supply sufficient air flow into an optical sensing part, as well as for completely blocking any external light [48]. Our study shows the influence of the air flow on PL quenching efficiency, which can be very useful for general gas sensing applications using polymers.

## Figures and Tables

**Table 1:**
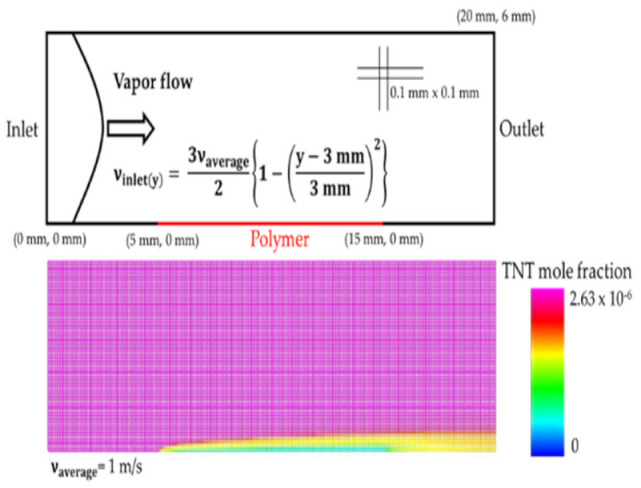
Simulation parameters. A simulation result of the TNT mole fraction is included.

Parameter	Value	Unit
Mesh size	0.1 × 0.1	mm^2^
Polymer surface concentration	3.20 × 10^−8^	kmol/m^2^
TNT diffusion coefficient	5.76 × 10^−6^	m^2^/s
O_2_ diffusion coefficient	1.76 × 10^−5^	m^2^/s
N_2_ diffusion coefficient	2.00 × 10^−5^	m^2^/s
TNT mole fraction	0.000000263	
O_2_ mole fraction	0.21	
N_2_ mole fraction	0.789999737	
Chemical reaction rate constant pre-exponential factor	10^6^	

## Supplementary Materials

The following supporting information can be downloaded at https://drive.google.com/drive/folders/14zbhJEvDLpJSTs4lxy-ZLjJ5KacWnlSY?usp=sharing, Supplementary Figure S1: Supplementary Figure 1.png; Supplementary Figure S2: Supplementary Figure 2.png.
