# Supplementary material for: Influence of Air Flow on Luminescence Quenching in Polymer Films towards Explosives Detection Using Drones
Source: Polymers (Basel). 2022 Jan 25;14(3):483. doi: 10.3390/polym14030483 (PMC8839006; doi:10.3390/polym14030483)
Supplement: Supplementary file 1 [file polymers-14-00483-s001.zip › polymers-1566535-supplementary.pdf]

polymers

MDPI

Supplementary Materials

Influence of Air Flow on Luminescence Quenching in Polymer Films Towards Explosives Detection Using Drones

Daegwon Noh [1,2], Emmanuel K. Ampadu [1,2] and Eunsoon Oh [1,2,*]

1 Department of Physics, Chungnam National University, 99 Daehak-ro Yuseong-gu, Daejeon 34134, Korea; fo1109@cnu.ac.kr (D.N.); ekampadu@cnu.ac.kr (E.K.A.)

2 Institute of Quantum Systems (IQS), Chungnam National University, 99 Daehak-ro Yuseong-gu, Daejeon 34134, Korea

* Correspondence: esoh@cnu.ac.kr

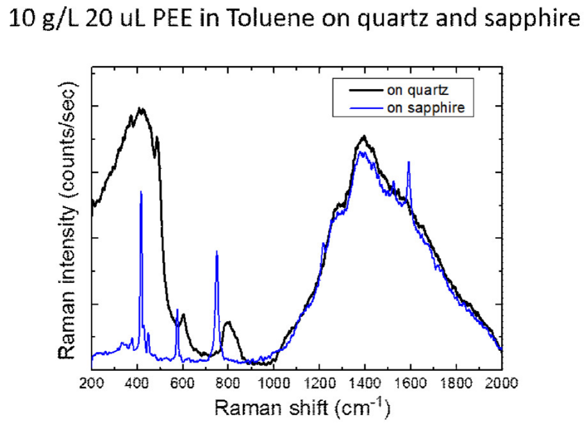

Figure S1. Room-temperature micro-Raman spectra of PEE polymer films on quartz and sapphire substrates with an excitation wavelength of 785 nm. A broad Raman peak at around 1400 cm^{-1} corresponds to a phonon mode at 170 meV.

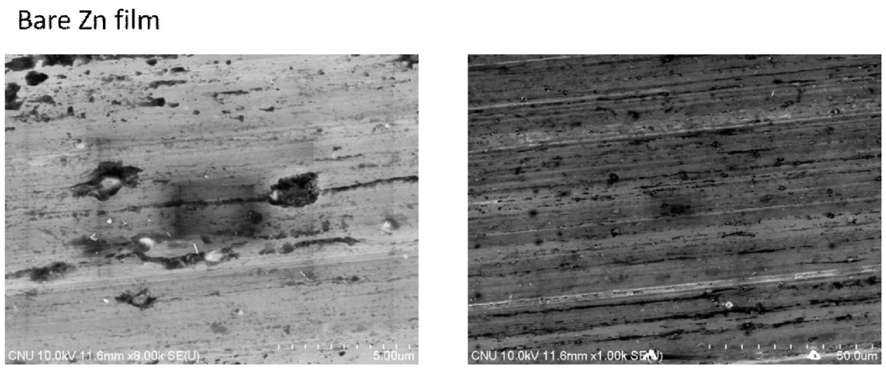

Figure S2. Surface SEM images of bare Zn substrates.
